# Evidence-based approach to the treatment of hidradenitis suppurativa/acne inversa, based on the European guidelines for hidradenitis suppurativa

**DOI:** 10.1007/s11154-016-9328-5

**Published:** 2016-02-01

**Authors:** Wayne Gulliver, Christos C. Zouboulis, Errol Prens, Gregor B. E. Jemec, Thrasivoulos Tzellos

**Affiliations:** 1 European Hidradenitis Suppurativa Foundation e.V, Dessau, Germany; 2 Faculty of Medicine, Memorial University of Newfoundland, St. John’s, Newfound land & Labrador Canada; 3 Departments of Dermatology, Venereology, Allergology and Immunology, Dessau Medical Center, Dessau, Germany; 4 Department of Dermatology, Erasmus MC, Rotterdam, The Netherlands; 5 Department of Dermatology, Roskilde Hospital, Health Sciences Faculty, University of Copenhagen, Copenhagen, Denmark; 6 Department of Dermatology, Faculty of Health Sciences, University Hospital of North Norway, Harstad, Troms Norway

**Keywords:** Hidradenitis suppurativa, Medical therapy, Surgical therapy, Evidence-based dermatology, Guidelines

## Abstract

Hidradenitis suppurativa/acne inversa (HS) is a chronic inflammatory skin disease characterized by painful, recurrent nodules and abscesses that rupture and lead to sinus tracts and scarring. To date, an evidence-based therapeutic approach has not been the standard of care and this is likely due to the lack of evidence based treatment guidelines. The purpose of this study was to promote a holistic evidence-based approach which implemented Level of Evidence and Strength of Recommendation for the treatment of HS. Based upon the European Dermatology Forumguidelines for the management of HS, evidence-based approach was explored for the treatment of HS. The diagnosis of HS should be made by a dermatologist or other healthcare professional with expert knowledge in HS. All patients should be offered adjuvant therapy as needed (pain management, weight loss, tobacco cessation, treatment of super infections, and application of appropriate dressings). The treating physician should be familiar with disease severity scores, especially Hurley staging, physician global assessment and others. The routine use of patient’reported outcomesincluding DLQI, itch and pain assessment (Visual Analogue Scale) is strongly recommended. The need for surgical intervention should be assessed in all patients depending upon type and extent of scarring, and an evidence-based surgical approach should be implemented. Evidence-based medical treatment of mild disease consists of topical Clindamycin 1 % solution/gel b.i.d. for 12 weeks or Tetracycline 500 p.o. b.i.d. for 4 months (LOE IIb, SOR B), for more widespread disease. If patient fails to exhibit response to treatment or for a PGA of moderate-to-severe disease, Clindamycin 300 p.o. b.i.d. with Rifampicin 600 p.o. o.d. for 10 weeks (LOE III, SOR C) should be considered. If patient is not improved, then Adalimumab 160 mg at week 0, 80 mg at week 2; then 40 mg subcutaneously weekly should be administered (LOE Ib, SOR A). If improvement occurs then therapy should be maintained as long as HS lesions are present. If the patient fails to exhibit response, then consideration of second or third line therapy is required. A growing body of evidence is being published to guide the treatment of HS. HS therapy should be based upon the evaluation of the inflammatory components as well as the scarring and should be directed by evidence-based guidelines. Treatment should include surgery as well as medical treatment. Future studies should include benefit risk ratio analysis and long term assessment of efficacy and safety, in order to facilitate long term evidence based treatment and rational pharmacotherapy.

## Introduction

Hidradenitis suppurativa/acne inversa (HS) is a chronic inflammatory skin disease characterized by painful, recurrent nodules and abscesses that rupture and lead to the formation of sinus tracts and scarring. HS has a psychologically meaningful and clinically significant negative effect on the patient’s quality of life. It is fairly common, affecting approximately 1 % of the population. Experience indicates that the patients are only diagnosed after long delays. This may be due to the previous orphan status of the disease. It is further speculated that an evidence-based therapeutic approach has therefore not been the standard of care to date. This status is likely reinforced by the lack of evidence based treatment guidelines. Based upon the Guidelines for HS treatment produced by the European Dermatology Forum [1], we are suggesting an evidence-based approach that includes Level of Evidence (LOE) and Strength of Recommendation (SOR) to produce a comprehensiveand rational approach for this debilitating and devastating chronic recurrent inflammatory disorder of the skin.

## Definition

HS is a chronic, inflammatory, recurrent, debilitating skin disease of the hair follicle that usually presents after puberty with painful, deep-seated, inflamed lesions in the body folds, most commonly the axillae, inguinal and anogenital regions, but can affect other areas as well (modified, Dessau definition, 1st International Conference on Hidradenitis suppurativa/Acne inversa, March 30-April 1, 2006, Dessau, Germany) [2, 3].

## Epidemiology

Studies which provide prevalence or incidence estimations have been performed under different settings (hospital versus population-based) and in different time periods. In addition, the applied diagnostic methods are varying (self-reported, medically assessed, diagnosis of treatments codes through automated requests in medical information systems) and, therefore, they led to an important variability regarding the prevalence of HS [4].

Using a health claims database, HS prevalence was estimated to be 0.05 % in the USA [5]. The authors reported that selection bias may have influenced results, since they included only health-insured subjects and, therefore, this prevalence rate may not be representative for the general US population. There may also be a classification bias as HS cases were only identified through drug reimbursement, leading to potential underestimations. Further analysis of the data implies important differences in the populations identified. The mean age of US cases was 38 years, i.e. older than the mean age of HS patients in European studies. Hence, young patients who have not asked for medical advice yet, may have been missed.

Two European studies have reported a prevalence of 1 % [6, 7]. The first was a French population-based investigation on self-declared cases in a representative sample of the French population (10,000) [6]. Even though selection bias is unlikely in this setting, a classification bias is likely due to self-declaration leading to potential over-estimation of the prevalence rate. Jemec et al. [7] assessed a sample of 599 Danish, clinically examined, unselected subjects and found a prevalence of 1 % (confidence intervals 0.4–2.2). Using a validated questionnaire in a population sample of 16 404 persons a possible prevalence of 2.1 % was suggested. In another study, Jemec found a prevalence of 4 % in young adult women [8]. As HS is mainly a disease of young adults with a female predominance, this result is not discordant with the figure of 1 % in the general population. These results are in discrepancy with the reported equal male to female ratio of the previous prospective study of the same authors [7].

The mean incidence of HS was assessed to be 6.0 per 100,000 person-years, evaluated through the Rochester Epidemiologic Project in an American county (Minnesota) with a population of about 144,000. Between 1968 and 2008 a two-fold incidence was calculated (4 to 10 per 100,000 person-years). This increase may be due to an increase in detection and coding of HS in the medical information system. The limitation of the study is its retrospective design. Moreover, a selection bias could have occurred due to recruitment through medical information system leading to a possible underestimation of incidence, since extrapolation of the data leads to the low prevalence of 0.08–0.20 % [9]. There also may be a classification bias due to missed diagnosis of mild early cases.

The discrepancies between European and American studies may be due to different methodologies but may also reflect actual differences in prevalence/incidence of HS or different diagnostic criteria with only the most severe cases having been reported in the USA.

### HS in children and adolescents

Data regarding HS in children and adolescents are scarce. HS can be seen in prepubertal age. Only 2 % of cases occur before the age of 11 years [10]. In a retrospective study of 855 patients, 7.7 % reported an onset of HS before the age of 13 years [11]. Early onset HS was associated with stronger genetic susceptibility and more widespread disease [11]. Recommendations for treatment in this age group are based only on case reports and extrapolation of treatments evaluated in clinical trials in adult population [12]. Finasteride, an antiandrogen, has been evaluated in a case series of early onset HS [13]. Most patients had endocrine co-morbidities, like polycystic ovary syndrome and precocious puberty. In all patients, finasteride, combined with antibiotics or other treatment modalities, facilitated remission and improved disease severity. Finasteride is recommended, especially in patients with refractory HS or with endocrine co-morbidities. Careful assessment of benefits and risks with its use is crucial in this age group.

Topical clindamycin twice daily for 3 months should be considered as first-line treatment option in mild to moderate HS. Systemic clindamycin plus rifampicine and tetracycline can be used, taking into careful account age restrictions, dosing differences, and possible side effects. Analgesics and corticosteroids can be considered to control pain and reduce inflammation. Adjuvant therapy and timely treatment of superinfections are essential. Scarring and Hurley III severity should be treated with surgical procedures. Negative pressure wound therapy in 30 HS patients (mean age 16 years) from a retrospective case series provide evidence that more 87 % of patients had successful outcomes with only 4 patients discontinuing because of side effects [14].

## Disease assessment

Objective disease assessment is a pre-requisite for the development of evidence based therapy. The Hurley classification of disease severity is the oldest and most commonly used of the several systems that have been suggested. The Hurley score is however a static score and not sufficiently responsive to change, particularly relating to the inflammatory component of HS [1]. In the past, the method of assessment was the Sartorius Scores, but in recent clinical research this has not been used as the primary endpoint. A newly developed physician rated assessment is the Hidradenitis Suppurativa Clinical Response (HiSCR) developed and validated and used as the primary endpoint in randomized control trials studying the use of adalimumab [15]. The HiSCR is not only a valid and meaningful endpoint for assessing HS treatment effectiveness in the inflammatory component of HS, but also its test-retest reliability, convergent validity, responsiveness and predictive validity were confirmed. The HiSCR is also significantly correlated with improvements in all physician-related measures (Hurley Stage, modified Sartorius Scores and HS Physicians Global Assessment), and patient reported outcomes (visual analogue pain scale, dermatology life quality index and work productivity and activity impairment questionnaire). HiSCR has been used to study the effect of adalimumab in HS and patients who continue responsiveness had significant improvements in both physician-related and patient-reported HS disease severity and impact [15].

## Methods

**Fig. 1:**
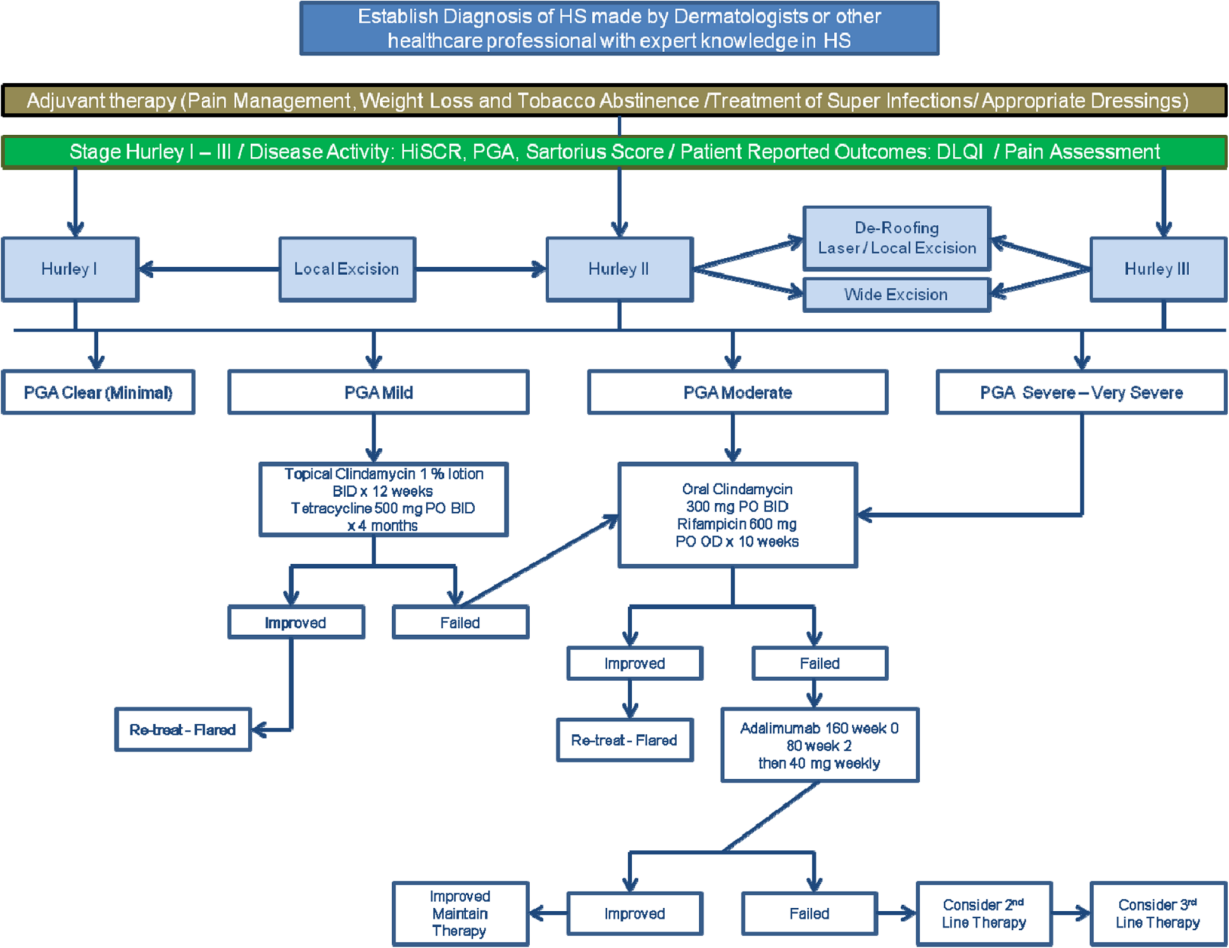
Algorithm

Based upon the recently published guidelines for hidradenitis suppurativa developed by the Guidelines Subcommittee of the European Dermatology Forum [1], a detailed review of all therapies was conducted. All therapeutic aspects of the treatment included in the Guidelines were reviewed and a category of evidence and strength of recommendation was developed for the first line therapy (see Table 1) which included topical Clindamycin, oral Clindamycin/Rifampicin, tetracycline and subcutaneous Adalimumab. A similar approach was taken for the second line therapies which included Zinc Gluconate, Resorcinol, Intralesional Corticosteroids, Infliximab, Acitretin and Etretinate. Third line therapies evaluated include Colchicine, Botulinum Toxin, Isotretinoin, Dapsone, Cyclosporine, hormones. Surgical therapies evaluated included excision or curettage of individual lesions, total excision of lesions surrounding hair-bearing skin, secondary intention healing, primary closure, reconstructive with skin grafting and NPWTreconstruction with flap, deroofing, carbon dioxide laser therapy, Nd:YAG laser therapy andintense pulsed light (IPL). Other aspects of treatment that were assessed include pain control and dressings (see Table 1). Based upon the Category of Evidence and Strength of Recommendation, an algorithm incorporating all aspects of HS therapy was developed (see Fig. 1). All therapeutic aspects with respect to HS therapy included in the Guidelines were assessed using the Grading of Recommendations Assessment and Evaluation (GRADE) methodology (see Table 2) [16]. At least 2 of the authors rated the medical and surgical treatments contained in the Guidelines.

**Table 1:** Category of Evidence/Strength of Recommendation Rating Scale

Therapy	Category of Evidence	Strength of Recommendation
1st Line		
Clindamycin (topical) ^1^	IIb	Possible B
Clindamycin/Rifampicin (oral) ^2^	III	C
Adalimumab (subcutaneous) ^3^	Ib	A
Tetracycline (oral)	IIb	B
Surgery		
Excision or Curettage of Individual Lesions	III	C
Total Excision of the Lesions and Surrounding Hair-Bearing Skin	IIb	B
Second Intention Healing	IIb	B
Primary Closure	III	C
Reconstruction with Skin Grafting & NPWT	III	C
Reconstruction with Flap Plasty	Ia/IIa	A/B
Deroofing	IV	D
Carbon Dioxide Laser Therapy	Ib	A
Nd:YAG Laser	Ib	A
IPL	IV	D
2nd Line		
Zinc Gluconate	III	C
Resorcinol	III	C
Intralesional Corticosteroids	IV	D
Systemic Corticosteroids	IV	D
Infliximab	Ib/IIa	B
Acitretin/Etretinate	III	C
3rd Line		
Colchicine	IV	D
Botulinum Toxin	IV	D
Isotretinoin	IV	D
Dapsone	IV	D
Cyclosporine	IV	D
Hormones	IV	D
Pain Control		
NSAIDS	IV	D
Opiates	IV	D
Dressings		
No studies have been published to date on the use of specific dressing or wound care methodology in HS. Choice of dressing is based on clinical experience.	IV	D

1. Single double-blind, placebo-controlled, randomized trial. Hurley stage 1–2.

2. Evaluated in case series.

3. Multiple prospective randomized, double-blind, placebo-controlled trials (Pioneer 1 and 2).

**Table 2:** Category or Evidence/Strength of Recommendation Rating Scale

Category of Evidence	Strength of Recommendation
Ia: Meta-analysis of randomized controlled trials Ib: Randomized controlled trial	A: Category I evidence
IIa: Controlled study without randomization IIb: Quasi-experimental study	B: Category II evidence or extrapolated from category I evidence
III: Non-experimental descriptive studies such as comparative, correlation and case-control studies	C: Category III evidence or extrapolated from category I or II evidence
IV: Expert committee reports or opinion or clinical experience of respected authorities, or both	D: Category IV evidence or extrapolated from category II or III evidence

Guyatt G, Oxman AD, et al. GRADE: An emerging consensus on rating quality of evidence and strength of recommendations. BMJ April 2008; 336: 924. Last accessed February 2014.

### First-line treatment options

#### Topical clindamycin

Clindamycin lotion 1 % is the only antibiotic that has been studied as topical agent. Its efficacy has been described in a double-blind, placebo-controlled, randomized trial in HS patients with Hurley stage I or mild stage II [17]. It is recommended as first line treatment option in patients with mild HS-PGA or localized Hurley I/mild Hurley II stage, especially when there are no deep inflammatory lesions (abscesses). The proposed dosing regimen is twice daily, for 3 months. If clinical response is not achieved after that treatment period, other treatment options must be considered (LOE IIb; SOR B).

#### Systemic antibiotics

##### Tetracycline

Tetracycline 500 mg b.i.d. has been tested and compared with topical clindamycin in a double-blind, randomized, controlled trial [18]. No significant difference was detected between the two treatment modalities. Tetracycline 500 mg b.i.d. is recommended as first line treatment option in patients with moderate HS-PGA or more widespread Hurley I/mild Hurley II stage, especially when there are no deep inflammatory lesions (abscesses), for up to 4 months. If clinical response is not achieved after that treatment period, other treatment modalities must be considered (LOE IIb; SOR B).

##### CLINDAMYCIN/RIFAMPICIN

Clindamycin 300 mg b.i.d. in combination with rifampicin 600 mg once daily or 300 mg b.i.d. has been evaluated in several case series [19]. This treatment combination is recommended as first-line treatment option in patients with moderate and severe HS-PGA or Hurley II stage, up to 10 weeks. If clinical responses not achieved after that treatment period, other treatment modalities must be considered (LOE III; SOR C).

#### Biologic agents

##### Adalimumab

Adalimumab sc 40 mg weekly, has been evaluated in a prospective, randomized, double-blind, placebo-controlled trial [20]. It influenced positively not only the primary outcome (HiSCR), but also pain (VAS pain score), DLQI and work productivity (TWPI score). Adalimumab is recommended as first-line treatment option in patients with moderate to severe HS who were unresponsive or intolerant to oral antibiotics. It should be administered as 160 mg at week 0, 80 mg at week 2 and 40 mg each week thereafter, starting from week 4.If clinical response with HiSCR is not achieved after 16 weeks of treatment, other treatment modalities must be considered (LOE Ib; SOR A) [21]. A preliminary report from a subsequent RCT supports the validity of the findings.

### Second-line treatment options

#### Biologic agents

##### Infliximab

Infliximab 5 mg/kg has been evaluated in a randomized, placebo-controlled, cross-over trial [22]. No significant difference in the >50 % improvement was detected (primary end point), while a significantly higher 25–50 % improvement rate was detected along with positive influence in DLQI and VAS pain score. Infliximab 5 mg/kg at week 0, 2, 6 and every 2 months thereafter for 12 weeks is recommended in patients with moderate to severe HS as second-line treatment option, only after failure of adalimumab. If clinical response is not achieved after 12 weeks of treatment, other treatment modalities must be considered (LOE IIa; SOR B).

##### Surgical treatment

Since non-surgical methods rarely result in lasting cure, surgical treatment is a quite common and accepted therapeutic modality for HS [1]. Surgical studies with classical evidenced-based evaluation of efficacy are generally sparse [23]. In agreement with the evidence paradigm of surgery, only large case series and studies reporting recurrence rates and a follow-up period of at least 1-year were therefore included in the evaluation.

### Radical surgical excision

Five studies looking at the role of radical excision (LOE IIb, SOR B) [24–28]. The type of surgery and margins were selected based on the body region and severity of the disease. A statistically significant relationship was shown between the efficacy of a procedure and the number and severity of body areas affected. The width of the excision and not the wound closure technique influenced the therapeutic outcome [25, 27, 29], whereas vacuum-assisted closure may accelerate the time-point of a delayed primary closure as shown by a retrospective study [30]. On the other hand, secondary intention healing revealed “good’ or “excellent” results in 27.3 % and 39.4 %, respectively, of 97 cases studied after 12 months of follow-up [31]. Buimer et al. [24] observed 41 % recurrences in 200 patients treated with radical surgery and closed by primary closure or skin graft. Bieniek et al. [25] described a complete recovery 59.7 % of 57 patients with 118 sites during a 2-year follow-up period. Bohn and Svensson [26] described the results of radical excision among 138 patients and follow-up from 3 to 21 years. In 38 of 116 skin-grafted patients (32.8 %) who completed a questionnaire, the disease recurred to some degree, and 14 of them required further operation. Van Rappard et al. [27] also experienced a 34 % recurrence rate in 92 primary closed surgically treated sites after 1–5 years follow-up. In contrast, Rompel and Petres [28] performed radical wide excision in 106 patients with a median postoperative follow-up time of 36 months and only a 2.5 % rate of recurrence within operated fields and wound infection in 3.7 % of patients. A retrospective study of 106 patients treated with radical excision, whereas 100 wounds were primary closed, 29 resurfaced with split skin grafts and 14 with local, fasciocutaneous or musculocutaneous flaps revealed 69.9 % recurrences after primary closure and no recurrences in the ‘graft’ and the ‘flap’ series [32]. In another retrospective study, 31 patients undergoing drainage procedures, limited regional surgery, and radical wide excisions confirmed the therapeutic advantage of radical excision showing 100 % recurrence after drainage, 42.8 % recurrence after limited excision, and 27 % recurrence after radical excision (*p* < 0.05; mean follow-up period 72 months) [33]. Similar results were reported by Wiltz et al. [34] in 43 patients with perianal involvement.

### Deroofing and skin-tissue-saving excision with electrosurgical peeling (STEEP)

An open study (SOR B) by van der Zee et al. [35] explored the efficacy of deroofing in 73 lesions; 83 % showed no recurrence during a median follow-up period of 34 months, and 17 % showed recurrence after a median follow-up period of 4.6 months. Postoperative bleeding in 1 patient was the only reported adverse event, and 90 % of patients responded that they would recommend the procedure to other individuals with HS. Blok et al. [36] evaluated retrospectively 113 patients, who underwent 482 operations (363 primary operations and 119 re-operations) with deroofing or STEEP. Relapses occurred after 29.2 % of primary operations in an average follow-up period of 43 months.

### Carbon dioxide LASER

Results of studies present controversial findings regarding the use of carbon dioxide LASER surgery in HS (LOE Ib, SOR A). Scanner assisted carbon dioxide LASER treatment under topical anesthesia was evaluated in a retrospective clinical study with 61 HS patients and 185 involved areas. A total number of 154 sessions led to a 1.1 % recurrence in a follow-up period of 1–19 years [37]. In most articles, healing by secondary intention was used. In another retrospective follow-up study in 34 patients with initial Hurley II stage, who were contacted by telephone after surgery for follow-up information, a mean healing time of 4 weeks was assessed with a recurrence rate of 11.8 % [38]. However, new lesions (separated from the initial surgical site by 5 cm) developed in 35.3 % of the patients, and 73.5 % of the patients had flares in an area other than the treated site. In a recent retrospective study involving 58 patients and the use of CO2 laser evaporation of lesions in patients with HS, 95 % of the patients reported some or great improvement, and 91 % would recommend the CO2 laser surgery. 29 % reported recurrence of HS lesions within the borders of the treated area [39].

### Nd:YAG LASER

Based on the assumption that HS starts in the hair follicle, the 1064-nm neodymium-doped yttrium aluminum garnet (Nd:YAG) laser was used in a prospective, randomized, controlled study on 22 patients with stage Hurley II to III HS [40, 41]. A series of 3 monthly laser sessions were performed. Treatment response was measured before each laser session and 1 month after the completion of laser treatment {HS Lesion, Area, and Severity Index (HS-LASI) scale}. A modification was made to include symptoms (erythema, edema, pain, and purulent discharge; modified HS-LASI, 0–3 scale]. The percentage change in HS severity after 3 months of treatment was −65.3 % over all anatomic sites, −73.4 % inguinal, −62.0 % axillary, and −53.1 % inframammary. For all anatomic sites combined and each individual anatomic site, the change in HS severity from baseline to month 3 was statistically significant at the treated sites (*p* < .02 for modified HS-LASI and HS-LASI), but not at the control sites (*p* > .05 for modified HS-LASI and HS-LASI) (LOE Ib, SOR A). After 4 monthly sessions, a right-left, treated-untreated comparison revealed an improvement of 72.7 % on the LASER-treated and 22.9 % on the control averaged over all anatomic sites (*p* < 0.05) [40] (LOE Ib, SOR A).

### Intense pulse light

A randomized study of 18 HS patients treated at one axilla, groin, or inframammary area with intense pulsed light twice weekly for 4 weeks, whereas the contralateral side received no treatment and served as a control, showed a significant improvement in the meanexamination score that was maintained at 12 months (*p* < 0.001 logistical regression analysis). The improvement was confirmed by independent assessment of clinical photographs (interrater reliability, 0.79; *p* < 0.001) [42] (LOE IV, SOR D).

## BENEFIT-RISK RATIO ANALYSIS

In order to go from evidence to recommendations that facilitate evidence-based daily clinical practice, the balance of desirable and undesirable outcomes of interest among alternative management strategies is becoming more and more important [43]. Benefit-risk ratio assessment with a structured approach is a becoming an outcome of great importance for authorities during drug regulatory decision-making as well [44].

A structured and appropriate benefit-risk ratio analysis can be performed only for the phase 2 trial of adalimumab, since that is the only randomized controlled trial with a thorough safety analysis [45]. Numbers needed to treat (NNT) for the primary outcome of HiSCR for the phase 2 data at week 16 was 4 (95 % CI: 2.1–10.7) [46], whereas numbers needed to harm (NNH) for any serious adverse event was 26. This favorable ratio of NNT to NHH, along with the fact that evidence from phase 2 trial indicates positive effect of adalimumab in other outcomes like DLQI, VAS pain score and TWPI, provide the basis to recommend adalimumab as the first-line treatment option in patients with moderate to severe HS who were unresponsive or intolerant to oral antibiotics.

## Discussion

Although we present an evidence-based approach to the diagnosis and treatment of HS, the first and most important step is that a dermatologist or other healthcare professional with expert knowledge in HS assess the patient and make the diagnosis, preferably at an early stage of the disease. Based upon the correct diagnosis and any other significant physical findings, appropriate investigations should be undertaken. HS is a clinical diagnosis and the need for biopsy, laboratory tests, cultures and investigations will be at the discretion of the examining physician and based on patient history and clinical findings. HS may be associated with comorbid diseases such as pyoderma, arthritis, obesity, IBD, anemia, lymphedema and metabolic syndrome [2]. Clinical evidence of any of these associated conditions should be appropriately investigated. Other important comorbid conditions include significant reduction in quality of life, depression, stigmatization and impairment of sexual health [1, 47, 48]. Like the other comorbid diseases this aspect of the disease needs to be assessed and managed appropriately.

Treatment recommendations for the special HS patients’ group such as children and adolescents were based only on case reports and extrapolation of clinical data in adults. Emphasis should be given to assessing comorbidities at baseline. There is an obvious need for clinical data of high level to be developed for this patient group.

As part of the holistic/broad-based approach all patients should be offered adjuvant therapy whether it be related to pain management, weight loss, tobacco cessation, treatment of super infections, or implementation of appropriate dressings [1]. The treating physician should also be familiar with disease severity scores, especially Hurley staging and Physician Global Assessment. Other disease severity scores that are important include HiSCR and Sartorius scores. Based on the adalimumab study it is suggested that the HS clinical response (HiSCR) [15] is currently the most appropriate clinical endpoint to assess treatment and effectiveness. As mentioned earlier, because of the significant impact on quality of life, patient reported outcomes are extremely important and these should include DLQI as well as pain assessment (Visual Analogue Scale).

With respect to surgical intervention, again this should be assessed in each individual patient and depending upon the type and extent of scarring, surgical procedures such as deroofing, laser, local excision or wide excision may be required.

Benefit-risk ratio structured assessment is needed to evaluate treatments, both short and long term. An initial primary benefit-risk ratio analysis was plausible only for adalimumab, for the first 16 weeks of treatment, which provides evidence of a favorable profile. Real life data from registries along with long term efficacy and safety data from clinical trial extension phases should be evaluated further, to assess long term benefit risk ratio and maintenance of treatment effect, in order to facilitate evidence based long term treatment.

## Conclusion

A growing body of evidence is being published to guide the treatment of HS. HS therapy should be based upon the evaluation of the inflammatory components as well as the scarring and should be directed by evidence-based therapy. Treatment should include surgery as well as medical treatment.
